# Dynamics of synchronous bursts in functionally coupled midbrain dopamine neurons driven by diverse excitatory inputs

**DOI:** 10.3389/fnsys.2026.1739960

**Published:** 2026-03-04

**Authors:** Meng-Jiao Chen

**Affiliations:** 1 School of Life Sciences, Leshan Normal University, Leshan, China; 2 Key Laboratory of Sichuan Institute for Protecting Endangered Birds in the Southwest Mountains, Leshan Normal University, Leshan, China

**Keywords:** burst transition, functional network of DA neurons, integrators, resonators, synchronous bursts

## Abstract

Phasic dopamine (DA) release is related to reward processing and addiction. The prevailing view posits that this DA release originates from synchronous bursts of DA neuron groups, rather than individual neuron bursts. However, the mechanism by which diverse excitatory inputs synergistically induce synchronous bursts remains unclear. In biophysically realistic networks with complex structure, the responses of functionally connected DA neurons to various excitatory inputs are examined. Activating NMDA receptors alone results in asynchronous bursts, while co-activating with muscarinic receptors significantly enhances burst synchronization. The synchronization trends display qualitatively similar characteristics across all tested topological networks, indicating that these synchronous bursts are universal. Research on a dual-node network reveals that inhibitory couplings, specifically the inward rectifying K^+^ currents activated by G protein linked to D2 receptors (D2-GIRK currents), participate in inducing synchronous bursts. A detailed analysis of decoupled DA neurons shows that these synchronous bursts are induced by transitioning bursts from integrator-like to resonator-like behavior, a process dependent on sufficient intracellular Ca^2+^ accumulation. NMDA receptors directly supply Ca^2+^, while muscarinic receptors indirectly provide Ca^2+^ by enhancing calcium-activated, nonspecific cation (CAN) current, causing depolarization, and activating L-type calcium channels. Therefore, simultaneous activation of both receptors is more effective in achieving the required Ca^2+^ accumulation than activating either receptor alone. These findings elucidate the mechanism by which diverse excitatory inputs work together to induce synchronous bursts, providing new insights into their inductions and regulations, potentially advancing our understanding of the physiological diversity of phasic DA releases and their addictive abnormalities.

## 1 Introduction

The ventral tegmental area (VTA) DA system is vital for reward-based neural activities, driving adaptive behaviors essential for survival and reproduction (Berke, 2018). Abnormalities in this system have been associated with conditions such as addiction. Traditionally, it was believed that changes in individual DA neuron electrical activity over time encode DA release, with tonic release associated with low-frequency spike firings and phasic release linked to high-frequency burst firings. However, this view is increasingly being challenged (Morales and Margolis, 2017), with experimental data supporting both homogeneity in individual DA neuron burst firing patterns (Eshel et al., 2016) and diversity in phasic releases in target regions (Parker et al., 2016). Several studies have reported that the synchronization of DA neuron populations is also involved in phasic release induction (Joshua et al., 2009; Li et al., 2011). As a result, an emerging perspective posits that spatial organization (synchronicity) of bursts is crucial for encoding robust phasic release (Beeler and Dreyer, 2019; Liu et al., 2021). Nonetheless, the precise mechanisms remain unclear.

Synchronization in neural networks depends on network structure, connections between neurons, and properties of neurons. Considering the impact of network structure on synchronization, DA networks exhibit stable complex network features, such as small-world (SW) properties, and cannot be regulated through D2 receptors (Miller et al., 2021). However, the role of network structure in synchronous bursts induced by excitatory inputs is still not fully understood.

Regarding the impact of connections between DA neurons upon synchronization, direct connections like gap junctions and synaptic links are relatively rare, whereas functional connections are prevalent. Activating D2 receptors by DA generates an inhibitory postsynaptic current, namely the D2-GIRK current (Beckstead et al., 2004). Theoretical studies show that inhibitory couplings typically lead to anti-phase (Li et al., 2019) or clustered synchronous bursts (Kim and Lim, 2019), which are less effective in producing phasic release compared to in-phase synchronous bursts. However, experimental evidence reveals that increased D2 receptors paradoxically facilitate phasic release (Kita et al., 2007; Jones and Fordahl, 2021). Theoretical studies on spiking networks reveal that sufficient inhibitory couplings can foster the synchronization of resonators (Izhikevich, 2010; Chik et al., 2004). This implies that strengthening inhibitory couplings through D2 receptors might similarly promote the synchronization of resonator bursts in DA neuronal networks.

Concerning the last category, akin to spikes, most bursts generally exhibit either a Saddle-Node (SN) or an Andronov-Hopf (AH) bifurcation structure of equilibria (Izhikevich, 2010, 2000), allowing them to function as integrators or resonators. Theoretical studies on spikes show that integrators and resonators respond differently to brief inhibitory perturbations. Integrators usually experience phase delays, whereas resonators often display both phase delays and phase advances (Izhikevich, 2010). These distinct responses result in significantly different synchronization properties, with resonator networks synchronizing more effectively than integrator networks. The properties of DA neurons are regulated by diverse external excitatory inputs through muscarinic and NMDA receptors (Wickham et al., 2015; Galaj et al., 2022). Studies on these bursts (Chen et al., 2022) reveal that, in the parameter plane defined by the intracellular Ca^2+^ concentration (*Ca*) and the gating variable *z* of SK current, the state-space trajectory of *Ca* and *z* dynamics intersects the SN bifurcation curve of equilibria during burst initiation. This finding aligns with earlier studies (Yu and Canavier, 2015; Oster et al., 2015), suggesting that these bursts function as integrators. However, as *Ca* and *z* increase, the bifurcation curve transitions from the SN to the AH curve via a Bogdanov-Takens (BT) bifurcation (Chen et al., 2022). This implies that the trajectory may intersect the AH curve during burst initiation, potentially allowing excitatory inputs to switch bursts from integrator to resonator behavior, thereby enhancing synchronization of the DA network.

Although activation of muscarine (Zhang et al., 2005) and NMDA receptors (Chergui et al., 1993) can both trigger bursts, co-infusion of carbachol, a muscarinic receptor agonist, with AP5, an NMDA receptor antagonist, into the VTA eliminated phasic release (Spanos et al., 2019), suggesting burst asynchronization; however, co-administration of carbachol and NMDA into the VTA restored phasic release (Spanos et al., 2019), indicating burst synchronization. These results imply that activating muscarinic and NMDA receptors separately tends to induce difficult-to-synchronize integrator bursts, whereas their simultaneous activation results in easy-to-synchronize resonator bursts, suggesting a synergistic effect between these receptors on inducing resonator bursts, though the exact mechanism is yet to be elucidated.

VTA DA neurons express TRPC channels, which mediate the CAN current (Klipec et al., 2016). Modulating the expression and function of these channels helps regulate DA neuron firing, and in turn, influences animal behavior (Wang et al., 2024). As G protein-coupled receptors, muscarinic receptors have the ability to modulate TRPC channel expression and function—a capability that has been confirmed in hippocampal pyramidal neurons (Tai et al., 2010). To substantiate speculations above, a functional network with SW characteristics is created, comprising 50 DA neurons bidirectionally connected by D2-GIRK currents. Each neuron features an *I*_*CAN*_ modulated by muscarinic receptors and an *I*_*NMDA*_ mediated by NMDA receptors (Chen et al., 2022). To examine the responses of DA neuron populations to diverse excitatory inputs, the spatiotemporal dynamics of networks are observed after separately and simultaneously activating NMDA and muscarinic receptors. To assess the impact of diverse excitatory inputs on synchronous bursts, synchronization index curves as a function of NMDA maximal conductance ${\bar{g}}_{N\,M\,D\,A}$ are compared before and after muscarinic receptor activation. To evaluate the influence of network structure on these bursts, synchronization index distribution diagrams across networks with different structures are compared. To investigate the role of inhibitory couplings in synchronous burst induction, D2-GIRK currents are evaluated across different network dynamics within a dual-node DA network. To understand how the local properties of DA neurons affect synchronous burst induction, a phase-plane analysis is performed on bursts of all conditions within a reduced model of decoupled DA neurons. To elucidate how muscarinic and NMDA receptors collaborate to transition bursts from integrator to resonator behavior, detailed fast-slow analyses are conducted on all electrical activities within a full model of decoupled DA neuron. To investigate the stability of the synchronization manifold induced by excitatory inputs, synchronization stability analyses of the systems under finite small perturbations are conducted.

Numerical results demonstrate that activating NMDA receptors alone shifts the DA network from a resting state to a burst asynchronization state. However, activating muscarinic receptors significantly improves the synchronization of NMDA receptor-induced bursts. These synchronous bursts are universal, as they exhibit qualitatively similar synchronization trends across all tested topologies. Further research on a dual-node DA network shows that inhibitory couplings participate in inducing synchronous bursts. Analyses on decoupled DA neurons reveal that transitioning to resonator bursts is crucial for synchronous burst induction, a process dependent on sufficient intracellular Ca^2+^ accumulation, which is more effectively achieved by simultaneously activating muscarinic and NMDA receptors. Stability analysis conducted under finite perturbations confirms that the synchronization induced by excitatory inputs is stable. These findings elucidate how diverse excitatory inputs collectively trigger synchronized bursts in midbrain DA neurons.

## 2 Materials and methods

### 2.1 The network model

To investigate how excitatory inputs trigger DA neuron populations, a bidirectionally connected SW network is developed by randomly rewiring the connections of a ring lattice with a probability of *p* = 0.1. The construction process includes the following steps: (1) Start with a ring of *N* = 50 DA neurons. (2) Connect each neuron to its 4 nearest neighbors (Miller et al., 2021). (3) Reconnect each connection to a randomly selected neuron with a probability of *p*. And our model of networked DA neurons (Equation 1) is as follow:


$$\dot{X_{i}}=F\,\left(X_{i},I_{i}^{N\,M\,D\,A},I_{i}^{C\,A\,N}\right)+\overset{N}{\underset{j=1}{\sum }}a_{i\,j}\,I_{i\,j}^{G\,I\,R\,K}\,H\,\left(X_{j}\right),$$ (1)

with *i*,*j* = 1,2,…,*N* representing the DA cell indices, and *X*_*i*_ denoting the state vector of the *i*th DA neuron. $\dot{X_{i}}=F\,\left(X_{i},I_{i}^{N\,M\,D\,A},I_{i}^{C\,A\,N}\right)$ describes the local dynamics of the *i*th DA neuron, with $I_{i}^{N\,M\,D\,A}$ and $I_{i}^{C\,A\,N}$ representing the diverse excitatory inputs to DA neuron *i*. The coupling relationship among DA neurons is captured by the adjacency matrix *A* = {*a*_*ij*_}, with *a*_*ij*_ = *a*_*ji*_ = 1 if DA neurons *i* and *j* are functionally interconnected, and *a*_*ij*_ = 0 otherwise. Meanwhile, the diagonal elements of *A* are set as *a*_ii_ = 0.

When DA neuron *j* is functionally interconnected with neuron *i* and becomes activated, it releases DA, which binds to D2 receptors on DA neuron *i*. These D2 receptors regulate GIRK channels through a biochemical cascade involving G proteins, resulting in a delayed and slow D2-GIRK current. The coupling function *H*(*X*_*j*_) is described by


$$H\,\left(X_{j}\right)=H\,\left(V_{j}\,\left(t-\tau \right)\right)=1.0/\left(1.0+e\,x\,p\,\left(-10.0\,\left(V_{j}\,\left(t-\tau \right)-\Theta_{s}\right)\right)\right),$$

with *V*_*j*_ denoting the membrane potential of DA neuron *j*, τ = 50 *ms* denoting the lag constant (Beckstead et al., 2004), and Θ_*s*_ = −20*mV* representing the coupling threshold (Otomo et al., 2020). And the coupling current $I_{i\,j}^{G\,I\,R\,K}$ is activated by switching *H*(*V*_*j*_(*t*−τ)) from 0 to 1, as described by Tian et al. (2022):


$$I_{i\,j}^{G\,I\,R\,K}={\bar{g}}_{i\,j}^{G\,I\,R\,K}\,r_{i}\,\left(V_{i}-E_{K}\right).$$

The dynamics of the gating variable *r*_*i*_ (Equation 2) are as follows:


$${\dot{r}}_{i}=\left(r_{\infty }\,\left(V_{i}\right)-r_{i}\right)/\tau_{r}\,\left(V_{i}\right),$$ (2)

with the functional equations for *r*_*i*_ given by


$$r_{\infty }\,\left(V_{i}\right)=\frac{1.0}{1.0+e\,x\,p\,\left(\frac{V_{i}+70.0}{20.0}\right)}+\frac{0.8}{1.0+e\,x\,p\,\left(\frac{V_{i}+70.0}{100.0}\right)},$$

$$\tau_{r}\,\left(V_{i}\right)=\frac{1.0}{0.006\,e\,x\,p\,\left(\frac{-V_{i}}{67.0}\right)+0.08\,e\,x\,p\,\left(\frac{V_{i}}{67.0}\right)}.$$

In line with previous studies (Leone et al., 2015), the coupling current $I_{i\,j}^{G\,I\,R\,K}$ is constrained to ensure a consistent total coupling current to each DA neuron: $I_{i\,j}^{G\,I\,R\,K}=I_{i}^{G\,I\,R\,K}/\kappa_{i\,n}$ (with κ_*in*_ representing the in-degree of DA neuron *i*). This constraint reflects biophysical reality, as experimental evidence shows that homeostatic plasticity mechanisms preserve network stability by preventing excessive or insufficient connections in DA neurons (Friedman et al., 2014; Zhang et al., 2019). In this study, the maximal conductance of the total coupling current received by each DA neuron is set at ${\bar{g}}_{i}^{G\,I\,R\,K}=0.005\,m\,S/c\,m^{2}$ (Courtney and Ford, 2014).

The local dynamics of DA neurons (Equations 3–9) are derived from previous research (Chen et al., 2022) as follows:


$$\begin{array}{cc} & C\,{\dot{V}}_{i}=-I_{i}^{N\,a}\,\left(V_{i},h_{i}\right)-I_{i}^{D\,R}\,\left(V_{i},n_{i}\right)-I_{i}^{l}\,\left(V_{i}\right)-I_{i}^{N\,a\,P}\,\left(V_{i}\right)-I_{i}^{K}\,\left(V_{i}\right)\\ & -I_{i}^{S\,K}\,\left(V_{i},z_{i}\right)-I_{i}^{C\,a\,L}\,\left(V_{i},d\,l_{i},f\,l_{i}\right)-I_{i}^{C\,A\,N}\,\left(V_{i},C\,a_{i}\right)-I_{i}^{N\,M\,D\,A}\,\left(V_{i}\right) \end{array},$$ (3)

$${\dot{h}}_{i}=\alpha_{h}\,\left(V_{i}\right)\,\left(1-V_{i}\right)-\beta_{h}\,\left(V_{i}\right)\,V_{i},$$ (4)

$${\dot{n}}_{i}=\alpha_{n}\,\left(V_{i}\right)\,\left(1-V_{i}\right)-\beta_{n}\,\left(V_{i}\right)\,V_{i},$$ (5)

$${\dot{d\,l}}_{i}=\left(d\,l_{\infty }\,\left(V_{i}\right)-d\,l_{i}\right)/\tau_{d\,l}\,\left(V_{i}\right),$$ (6)

$${\dot{f\,l}}_{i}=\left(f\,l_{\infty }\,\left(V_{i}\right)-f\,l_{i}\right)/\tau_{f\,l}\,\left(V_{i}\right),$$ (7)

$${\dot{z}}_{i}=\left(z_{\infty }\,\left(C\,a_{i}\right)-z_{i}\right)/\tau_{z},$$ (8)

$${\dot{C\,a}}_{i}=\varepsilon \,\left(-\kappa_{1}\,I_{i}^{N\,M\,D\,A}\,\left(V_{i}\right)-I_{i}^{C\,a\,L}\,\left(V_{i},d\,l_{i},f\,l_{i}\right)-\kappa_{2}\,\left(C\,a_{i}-C\,a_{b\,a\,s\,a\,l}\right)\right),$$ (9)

where, for the *i*th DA neuron, *V*_*i*_ is the membrane potential, *h*_*i*_ is the inactivation variable for Na^+^ current ($I_{i}^{N\,a}$); *n*_*i*_ is the activation variable for delayed-rectifier K^+^ current ($I_{i}^{D\,R}$); *dl*_*i*_ and *fl*_*i*_ are the activation variable and inactivation variable for L-type Ca^2+^ current ($I_{i}^{C\,a\,L}$), respectively; *z*_*i*_ is the activation variable for SK current ($I_{i}^{S\,K}$); *Ca*_*i*_ is the intracellular Ca^2+^ concentration. $I_{i}^{l}$ is the leak current, $I_{i}^{N\,a\,P}$ is the persistent Na^+^ current, $I_{i}^{K}$ is the generic persistent K^+^ current, $I_{i}^{N\,M\,D\,A}$ is the NMDA current, and $I_{i}^{C\,A\,N}$ is the muscarinic receptor-modulated CAN current.

All currents are represented by chord-conductance equations, as shown below:


$$I_{i}^{N\,a}={\bar{g}}_{N\,a}\cdot m_{\infty }\,{\left(V_{i}\right)}^{3}\cdot h_{i}\cdot \left(V_{i}-E_{N\,a}\right),$$

IiD⁢R=g¯D⁢R⋅ni4⋅(Vi-EK),

$$I_{i}^{l}={\bar{g}}_{l}\cdot \left(V_{i}-E_{l}\right),$$

$$I_{i}^{N\,a\,P}={\bar{g}}_{N\,a\,p}\cdot q_{\infty }\,\left(V_{i}\right)\cdot \left(V_{i}-E_{N\,a}\right),$$

$$I_{i}^{K}={\bar{g}}_{K}\cdot p_{\infty }\,\left(V_{i}\right)\cdot \left(V_{i}-E_{K}\right),$$

IiS⁢K=g¯S⁢K⋅zi2⋅(Vi-EK)

$$I_{i}^{C\,a\,L}={\bar{g}}_{C\,a\,L}\cdot d\,l_{i}\cdot f\,l_{i}\cdot \left(V_{i}-E_{C\,a}\right),$$

$$I_{i}^{C\,A\,N}={\bar{g}}_{C\,A\,N}\cdot o_{\infty }\,\left(C\,a_{i}\right)\cdot \left(V_{i}-E_{C\,A\,N}\right),$$

$$I_{i}^{N\,M\,D\,A}={\bar{g}}_{N\,M\,D\,A}\cdot s_{\infty }\,\left(V_{i}\right)\cdot \left(V_{i}-E_{N\,M\,D\,A}\right).$$

And functional equations for all gating variables are


$$m_{\infty }\,\left(V_{i}\right)=0.5\,\left[1.0-t\,a\,n\,h\,\left(\frac{14.0+V_{i}}{-11.9}\right)\right],$$

$$\alpha_{h}\,\left(V_{i}\right)=0.025\,\left[1.0+t\,a\,n\,h\,\left(\frac{42.0+V_{i}}{-15.0}\right)\right],$$

$$\beta_{h}\,\left(V_{i}\right)=0.55\,\left[1.0-t\,a\,n\,h\,\left(\frac{10.0+V_{i}}{-8.5}\right)\right],$$

$$\alpha_{n}\,\left(V_{i}\right)=0.5\,\left[1.0-t\,a\,n\,h\,\left(\frac{100.0-V_{i}}{80.0}\right)\right],$$

$$\beta_{n}\,\left(V_{i}\right)=1.0\,\left[1.0+t\,a\,n\,h\,\left(\frac{30.0+V_{i}}{-10.0}\right)\right],$$

$$d\,l_{\infty }\,\left(V_{i}\right)=\frac{1.0}{1.0+e\,x\,p\,\left(\frac{8.21-V_{i}}{11.89}\right)},$$

$$\tau_{d\,l}\,\left(V_{i}\right)=\frac{77.0}{1.0+e\,x\,p\,\left(\frac{39.0+V_{i}}{2.6}\right)}+17.0,$$

$$f\,l_{\infty }\,\left(V_{i}\right)=\frac{1.0}{1.0+e\,x\,p\,\left(\frac{30.0+V_{i}}{28.74}\right)},$$

$$\tau_{f\,l}\,\left(V_{i}\right)=\frac{1.0}{1.0+e\,x\,p\,\left(\frac{40.0+V_{i}}{3.0}\right)}+0.78,$$

z∞⁢(C⁢ai)=C⁢ai4C⁢ai4+κS⁢K4,q∞⁢(Vi)=1.11.0+e⁢x⁢p⁢(Vi+50.0-3.0),

$$p_{\infty }\,\left(V_{i}\right)=\frac{1.0}{1.0+e\,x\,p\,\left(\frac{V_{i}+15.0}{-7.0}\right)},o_{\infty }\,\left(C\,a_{i}\right)=\frac{1.0}{1.0+e\,x\,p\,\left(\frac{C\,a_{i}-0.3}{-0.05}\right)},$$

$$s_{\infty }\,\left(V_{i}\right)=\frac{1.0}{1.0+C_{M\,g}\,e\,x\,p\,\left(-0.062\,V_{i}\right)}.$$

All parameter values are given in Table 1. The values of ${\bar{g}}_{C\,A\,N}$ and ${\bar{g}}_{N\,M\,D\,A}$ are uniformly distributed over the intervals [0.9, 2.5] *mS/cm^2^* and [0, 0.025] *mS/cm^2^*, respectively.

**TABLE 1:** The parameter values.

Parameter	Value	Parameter	Value	Parameter	Value	Parameter	Value
${\bar{g}}_{N\,a}$	250.0*mS*/*cm*^2^	${\bar{g}}_{S\,K}$	3.5*mS*/*cm*^2^	*E*_*CAN*_	0*mV*	*C*	1.0μ*F*
${\bar{g}}_{D\,R}$	5.0*mS*/*cm*^2^	${\bar{g}}_{N\,M\,D\,A}$	0*mS*/*cm*^2^	*E*_*NMDA*_	0*mV*	τ_*z*_	100.0*ms*
${\bar{g}}_{K}$	0.4*mS*/*cm*^2^	${\bar{g}}_{G\,I\,R\,K}$	0.005*mS*/*cm*^2^	ε	0.0025	*C*_*Mg*_	0.5μ*M*
${\bar{g}}_{N\,a\,P}$	0.002*mS*/*cm*^2^	*E*_*Na*_	55.0*mV*	κ_1_	0.3	Θ_*S*_	−20*mV*
${\bar{g}}_{l}$	0.015*mS*/*cm*^2^	*E*_*K*_	−90.0*mV*	κ_2_	2	τ	50.0*ms*
${\bar{g}}_{C\,a\,L}$	0.075*mS*/*cm*^2^	*E*_*Ca*_	100.0*mV*	κ_*SK*_	0.3	*k*_*in*_	4
${\bar{g}}_{C\,A\,N}$	0.9*mScm*^2^	*E*_*l*_	−50.0*mV*	*Ca*_*basal*_	0.005		

All parameters are directly adopted from Chen et al. (2022), except for the parameters marked in gray. The reference literature for the gray parameters is detailed in Table 2.

**TABLE 2:** Source literatures for the gray parameters in Table 1.

Parameter	Range	References
${\bar{g}}_{G\,I\,R\,K}$	0.001∼0.01	Courtney and Ford, 2014
τ	50∼60 ms	Beckstead et al., 2004
κ_*in*_	3.97∼5.24	Miller et al., 2021
Θ_*S*_	−20 mV	Otomo et al., 2020

### 2.2 Synchronization index

To evaluate burst synchronization, the order parameter *R* is utilized as follows (Sun and Xue, 2018):


$$R=\frac{\left|\sum_{j=1}^{N}e^{i\,\varphi_{j}\,\left(t\right)}\right|}{N},$$

with *N* = 50 representing the total number of networked DA neurons, and φ_*j*_(*t*) denoting the burst phase of the *j*th DA neuron at the time *t*, this can be expressed as


$$\varphi_{j}\,\left(t\right)=\frac{2\,\pi \,\left(t-T_{j,k}\right)}{T_{j,k+1}-T_{j,k}},T_{j,k}\leq t\leq T_{j,k+1},$$

where *T*_*j*,*k*_ represents the onset time of the *k*th burst of DA neuron *j*. A higher *R* value signifies greater burst synchronization. Specifically, *R* values below 0.4 indicate asynchronization, between 0.4 and 0.8 indicate moderate synchronization, between 0.8 and 0.99 indicate near synchronization, and between 0.99 and 1 indicate full synchronization (Xu et al., 2021).

### 2.3 Phase-plane analysis and fast-slow analysis

Each decoupled DA neuron operates on a slow-fast system, where the (*V*, *h*, *n*, *dl*, *fl*)-equations constitute a 5-D fast “spiking” subsystem, and the (*Ca*, *z*)-equations make up a 2-D slow “bursting” subsystem. The CAN current, controlled by *Ca*, and the SK current, gated by *z*, together produce a slow voltage oscillation. When this oscillation exceeds the firing threshold (*V*_*th*_) of the fast subsystem, a series of action potentials is initiated, namely a burst.

To explore the mechanism of synchronous burst induction, the decoupled DA neuron is reduced into a 3-D model comprising (*V*, *Ca*, *z*) by setting the fast gating variables (*h*, *n*, *fl*, *dl*) to their steady states. The resulting reduced model (Equations 10–12) is:


$$\begin{array}{cc} & C\,\dot{V}=-I_{N\,a}\,\left(V\right)-I_{D\,R}\,\left(V\right)-I_{l}\,\left(V\right)-I_{N\,a\,P}\,\left(V\right)-I_{K}\,\left(V\right)\\ & -I_{S\,K}\,\left(V,z\right)-I_{C\,a\,L}\,\left(V\right)-I_{C\,A\,N}\,\left(V,C\,a\right)-I_{N\,M\,D\,A}\,\left(V\right) \end{array},$$ (10)

$$\dot{C\,a}=\varepsilon \,\left(-\kappa_{1}\,I_{N\,M\,D\,A}\,\left(V\right)-I_{C\,a\,L}\,\left(V\right)-\kappa_{2}\,\left(C\,a-C\,a_{b\,a\,s\,a\,l}\right)\right),$$ (11)

$$\dot{z}=\left(z_{\infty }\,\left(C\,a\right)-z\right)/\tau_{z}.$$ (12)

Phase-plane analysis of each burst is conducted within the reduced model. The equilibrium bifurcation structure of bursts is defined by the intersection of *Ca* nullclines and *V* nullclines at *V*_*th*_. A tangential intersection indicates a SN bifurcation, characteristic of integrator bursts. A direct intersection, however, signifies an AH bifurcation, indicative of resonator bursts.

To explore how diverse excitatory inputs work together to induce resonator bursts, a fast-slow analysis of electrical activities within the full DA model is conducted, with special attention paid to the differences in state-space distributions of *Ca* and *z* dynamics during the initiation of different bursts.

### 2.4 Stability analysis of synchronization with finite perturbations

To examine the stability of synchronization, how the system’s synchronization behaves is analyzed when subjected to finite perturbations. The specific idea is: once DA neuronal network has achieved a stable synchronization, small perturbations will be randomly introduced into a subset of DA cells, and then observe whether the system can recover and re-establish synchronization (Arenas et al., 2008).

In the two-node DA network, the occurrence time of the first spike within each burst is used to represent the burst occurrence time, denoted as $t_{1}^{\left(n\right)}$ and $t_{2}^{\left(n\right)}$ respectively. Then, the phase difference between bursts of coupled DA cells is calculated using the formula:


$$\Psi_{n}=\frac{\left|t_{2}^{\left(n\right)}-t_{1}^{\left(n\right)}\right|}{T_{b\,u\,r\,s\,t}},$$

where *T*_*burst*_ represents the bursting period. The variation process of Ψ_*n*_ with respect to the number of bursting periods *n* after finite perturbations is used to characterize synchronization stability. If the Ψ_*n*_−*n* curve decays monotonically or oscillatorily to the pre-perturbation level, this will intuitively confirm the stability of synchronization.

In complex networks composed of 50 DA neurons, the decay and recovery processes of the order parameter *R*(*t*) following finite perturbations are directly used to characterize the stability of synchronization. By comparing the relaxation time and recovery rate of *R*(*t*), the faster the recovery, the more stable the synchronization.

Numerical simulations are executed using Microsoft Visual C++, phase-plane analysis is performed with MATLAB, and fast-slow analysis is carried out using MatCont. Initial values for each DA neuron are randomly assigned, and differential equations are solved using the fourth-order Runge-Kutta method with a step size of 0.01 *ms*.

## 3 Results

### 3.1 Co-activation of NMDA and muscarinic receptors induce synchronous bursts

**FIGURE 1:**
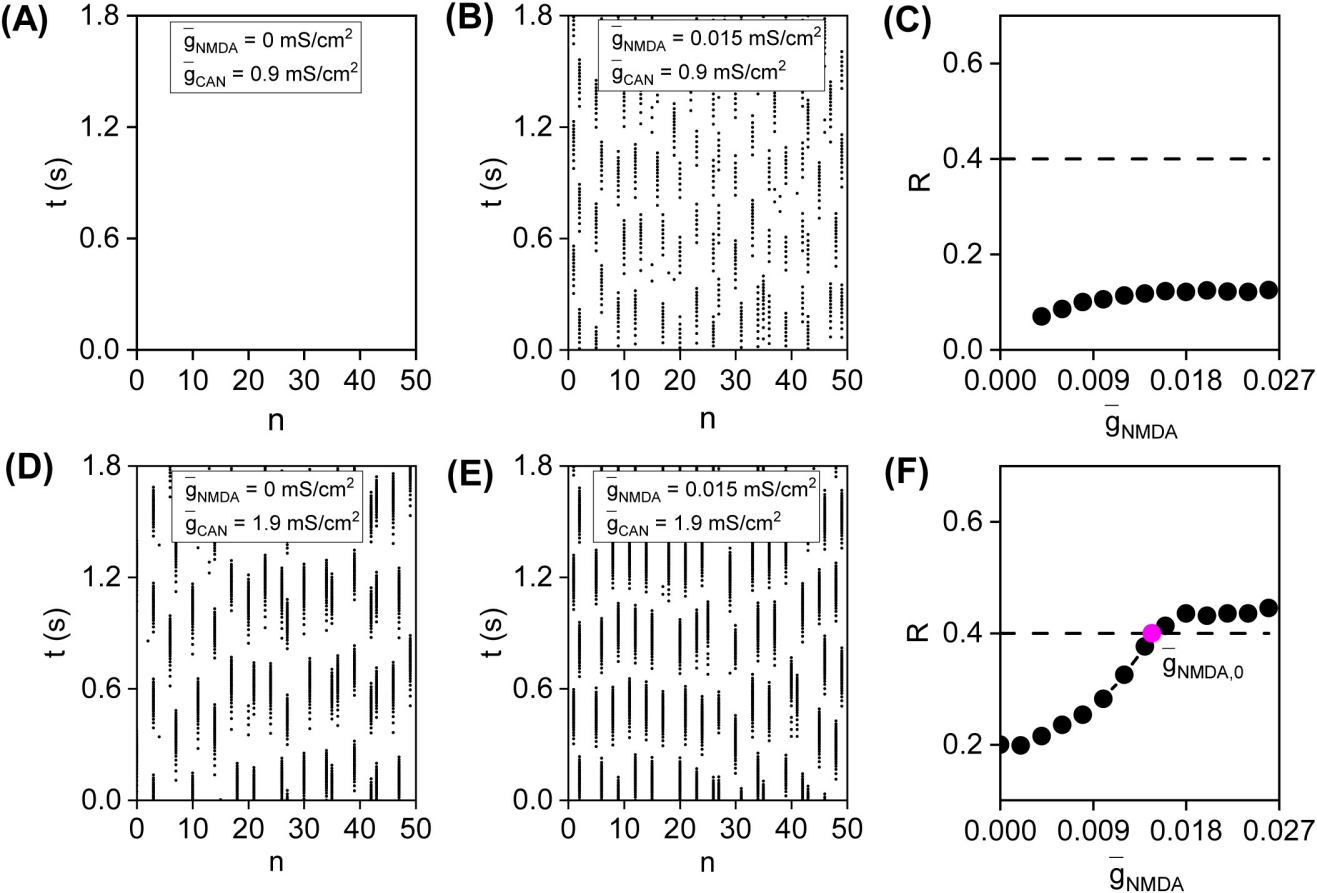
Synchronous bursts induced by simultaneous activation of NMDA and muscarinic receptors in a small-world (SW) DA network. Maintaining ${\bar{g}}_{C\,A\,N}$ of each DA neuron at 0.9*mS*/*cm*^2^ to simulate conditions before muscarinic receptor activation, the spatiotemporal dynamics of DA network before (${\bar{g}}_{N\,M\,D\,A}=0\,m\,S/c\,m^{2}$) **(A)** and after (${\bar{g}}_{N\,M\,D\,A}=0.015\,m\,S/c\,m^{2}$) **(B)** NMDA receptor activation. **(C)** the variation of order parameter *R* as a function of ${\bar{g}}_{N\,M\,D\,A}$ before muscarinic receptor activation. Increasing ${\bar{g}}_{C\,A\,N}$ to 1.9*mS*/*cm*^2^ to simulate conditions after muscarinic receptor activation, the network dynamics before (${\bar{g}}_{N\,M\,D\,A}=0\,m\,S/c\,m^{2}$) **(D)** and after (${\bar{g}}_{N\,M\,D\,A}=0.015\,m\,S/c\,m^{2}$) **(E)** NMDA receptor activation. **(F)** *R*-${\bar{g}}_{N\,M\,D\,A}$ curve after muscarinic receptor activation. The dashed lines denote moderate synchronization threshold of *R* = 0.4, and the magenta dot denotes the ${\bar{g}}_{N\,M\,D\,A}$ corresponding to moderate synchronization threshold of *R* = 0.4, designated as ${\bar{g}}_{N\,M\,D\,A,0}$.

To investigate the impact of NMDA receptors on DA network’s behavior, muscarinic receptor-modulated CAN maximal conductance ${\bar{g}}_{C\,A\,N}$ of each DA neuron is maintained at 0.9*mS*/*cm*^2^, representing the state before muscarinic receptor activation. The spatiotemporal dynamics of the network are observed by adjusting NMDA maximal conductance ${\bar{g}}_{N\,M\,D\,A}$ within each DA neuron. With ${\bar{g}}_{N\,M\,D\,A}$ set to 0*mS*/*cm*^2^, reflecting the state before NMDA receptor activation, the network dynamics after a 2.5 sec transient period are illustrated in Figure 1A, with all neurons at resting membrane potentials, indicating that DA network is in a resting state. Increasing ${\bar{g}}_{N\,M\,D\,A}$ to 0.015 *mS*/*cm*^2^ to simulate NMDA receptor activation, as depicted in Figure 1B, results in burst firings in all DA neurons, but these bursts seem asynchronous.

To illustrate how the network dynamics vary with ${\bar{g}}_{N\,M\,D\,A}$ before muscarinic receptor activation, the order parameter *R* is plotted against ${\bar{g}}_{N\,M\,D\,A}$ in Figure 1C. As illustrated, increasing ${\bar{g}}_{N\,M\,D\,A}$ leads to a gradual rise in *R*, yet it never exceeds the synchronization threshold of 0.4. This indicates that DA network remains in a state of burst asynchronization throughout the ${\bar{g}}_{N\,M\,D\,A}$ range, demonstrating that activating NMDA receptors alone is insufficient to induce synchronous bursts.

Previous research showed that, in addition to NMDA receptors, muscarinic receptors are essential for natural reward stimuli to elicit phasic release (Wickham et al., 2015). This finding highlights the need to investigate the impact of muscarinic receptors on NMDA receptor-induced bursts. As depicted in Figure 1D, increasing the CAN maximal conductance ${\bar{g}}_{C\,A\,N}$ to 1.9*mS*/*cm*^2^ to mimic muscarinic receptor activation results in the re-emergence of asynchronous bursts prior to NMDA receptor activation (${\bar{g}}_{N\,M\,D\,A}=0\,m\,S/c\,m^{2}$). However, upon NMDA receptor activation (${\bar{g}}_{N\,M\,D\,A}=0.015\,m\,S/c\,m^{2}$), as depicted in Figure 1E, the bursts become more synchronized.

Following muscarinic receptor activation, the $R-{\bar{g}}_{N\,M\,D\,A}$ curve is explored again. As depicted in Figure 1F, *R* rises from below 0.4 to between 0.4 and 0.8 as ${\bar{g}}_{N\,M\,D\,A}$ increases. As per Xu et al. (2021), the ${\bar{g}}_{N\,M\,D\,A}$ value corresponding to the synchronization threshold *R* of 0.4 is designated as ${\bar{g}}_{N\,M\,D\,A,0}$ (Figure 1F, magenta dot). For ${\bar{g}}_{N\,M\,D\,A}$ values smaller than ${\bar{g}}_{N\,M\,D\,A,0}$, *R* remains below 0.4, indicating the DA network is in a state of burst asynchronization. However, for ${\bar{g}}_{N\,M\,D\,A}$ values greater than ${\bar{g}}_{N\,M\,D\,A,0}$, *R* increases from below 0.4 to between 0.4 and 0.8, suggesting the DA network shifts to a state of moderate burst synchronization. These results highlight that muscarinic receptor-modulated CAN current promotes NMDA receptor-induced bursts synchronizing.

**FIGURE 2:**
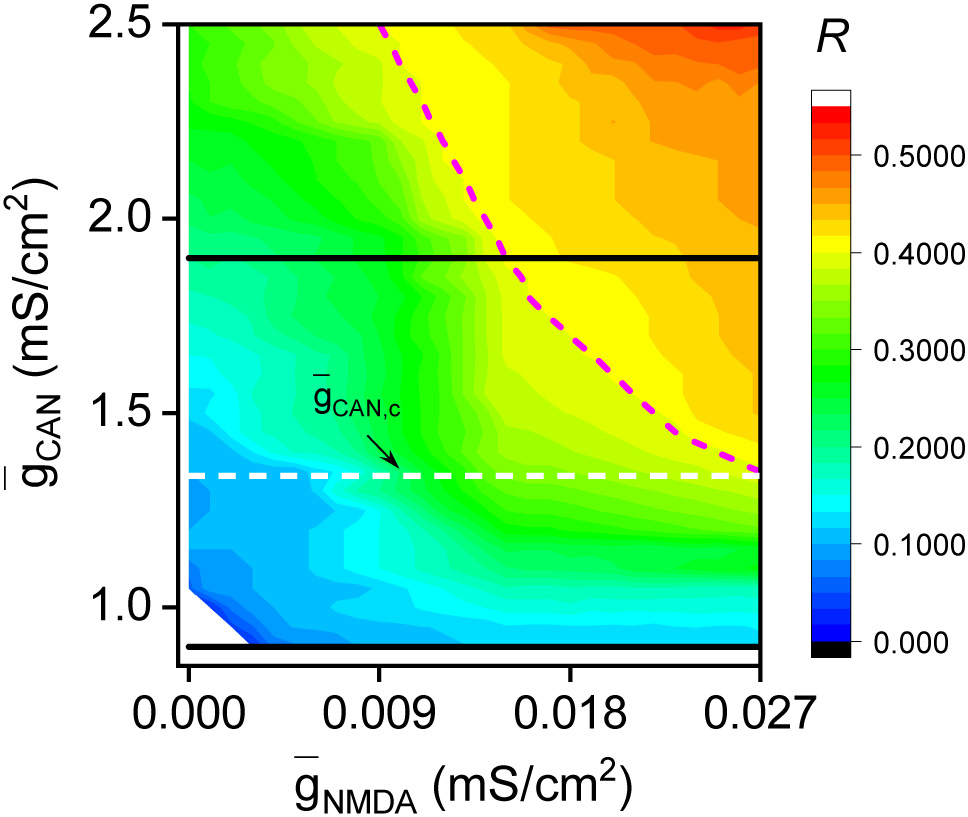
*R* distribution diagram on the parameter space (${\bar{g}}_{C\,A\,N}$, ${\bar{g}}_{N\,M\,D\,A}$). When muscarinic receptor-modulated calcium-activated, nonspecific cation (CAN) maximal conductance is high (${\bar{g}}_{C\,A\,N}>{\bar{g}}_{C\,A\,N,c}\approx 1.31$), with the increase of ${\bar{g}}_{C\,A\,N}$, ${\bar{g}}_{N\,M\,D\,A,0}$ monotonically decreases, demonstrating that the moderate burst synchronization region gradually expands to the left, covering a larger portion of the ${\bar{g}}_{N\,M\,D\,A}$ range. When CAN maximal conductance is low (${\bar{g}}_{C\,A\,N}<{\bar{g}}_{C\,A\,N,c}$), ${\bar{g}}_{N\,M\,D\,A,0}$ is nonexistent, indicating the burst asynchronization region occupying the entire ${\bar{g}}_{N\,M\,D\,A}$ range. The horizontal lines denote *R*-${\bar{g}}_{N\,M\,D\,A}$ curves shown in Figures 1C, F, respectively. The white dashed line denotes the critical CAN maximal conductance ${\bar{g}}_{C\,A\,N,c}$. The magenta dashed curve denotes the boundary composed by ${\bar{g}}_{N\,M\,D\,A,0}$.

To globally understand the impact of muscarinic receptors on NMDA receptor-induced bursts, a detailed diagram of *R* distribution across a specific region in the two-dimensional parameter space (${\bar{g}}_{C\,A\,N}$, ${\bar{g}}_{N\,M\,D\,A}$) is scanned. As shown in Figure 2, when the CAN maximal conductance is low (${\bar{g}}_{C\,A\,N}<{\bar{g}}_{C\,A\,N,c}\approx 1.31$), ${\bar{g}}_{N\,M\,D\,A,0}$ is nonexistent, indicating the burst asynchronization region occupying the entire ${\bar{g}}_{N\,M\,D\,A}$ range. And when the CAN maximal conductance is high (${\bar{g}}_{C\,A\,N}>{\bar{g}}_{C\,A\,N,c}$), ${\bar{g}}_{N\,M\,D\,A,0}$ gradually decreases as ${\bar{g}}_{C\,A\,N}$ increases (Figure 2, magenta dashed line). Therefore, the moderate burst synchronization region expands gradually to the left, occupying a larger portion of the ${\bar{g}}_{N\,M\,D\,A}$ range. This indicates that higher levels of muscarinic receptor-modulated CAN current significantly enhance the possibility of NMDA receptor-induced bursts synchronizing, indicating a complex interaction between two receptors in promoting synchronous bursts.

### 3.2 Synchronous bursts induced by excitatory inputs are universal

**FIGURE 3:**
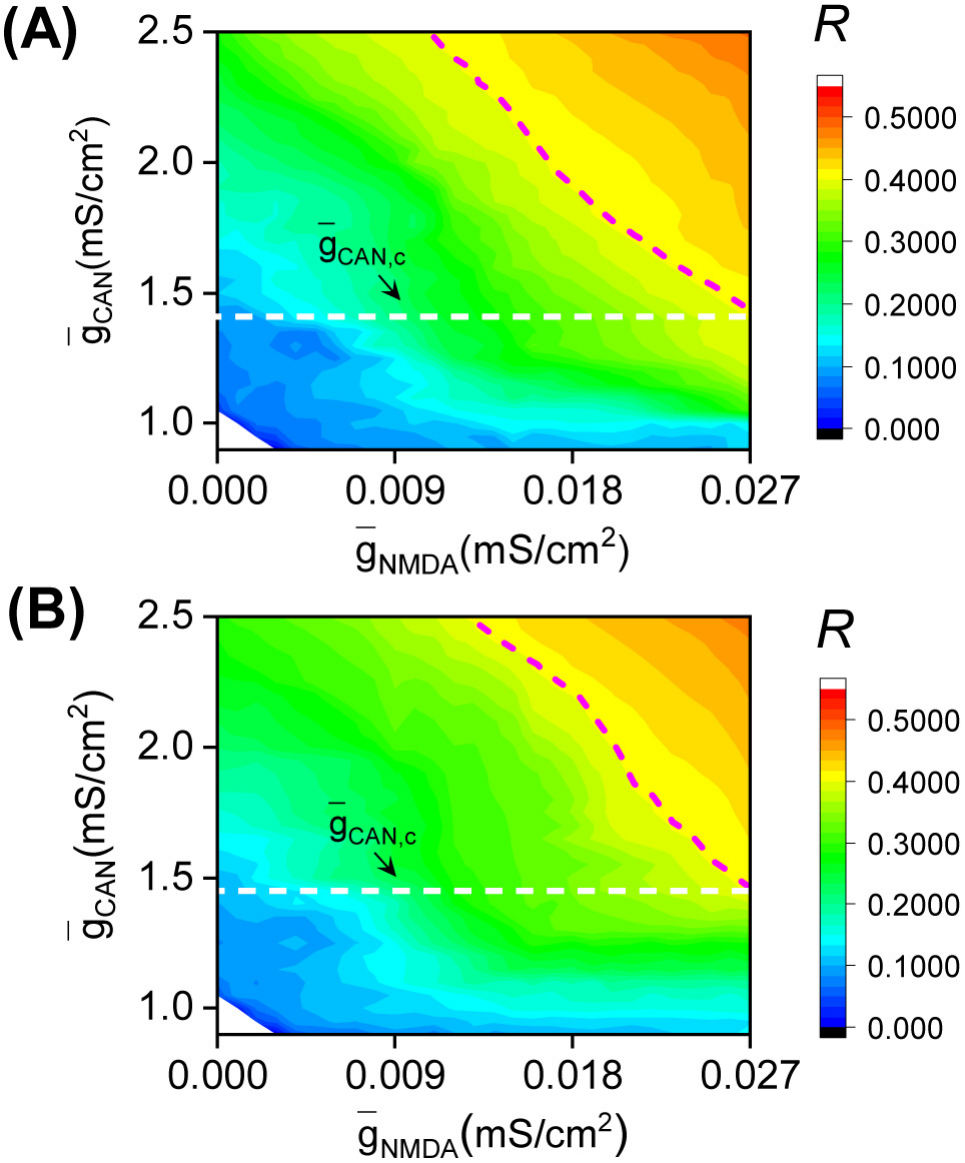
*R* distribution diagrams for the Barabási-Albert (BA) **(A)** and Erdös-Rényi (ER) **(B)** networks. The size and the number of links of the networks are the same to that of SW network used in Figure 2. For the BA (ER) network, the moderate burst synchronization region gradually expands to the left as ${\bar{g}}_{C\,A\,N}$ increases from ${\bar{g}}_{C\,A\,N,c}\approx 1.38$ (${\bar{g}}_{C\,A\,N,c}\approx 1.42$), and the burst asynchronization region occupies the entire ${\bar{g}}_{N\,M\,D\,A}$ range in the region ${\bar{g}}_{C\,A\,N}<{\bar{g}}_{C\,A\,N,c}$. The white dashed lines denote the critical CAN maximal conductance ${\bar{g}}_{C\,A\,N,c}$. The magenta dashed curves denote the boundaries composed by ${\bar{g}}_{N\,M\,D\,A,0}$.

Similar *R* distribution diagrams are observed for other network structures. Figure 3A illustrates the *R* distribution for the Barabási-Albert (BA) network, which is constructed through the preferential attachment of newly added nodes to those with rich connections. The BA network’s size and number of links match those of the SW network depicted in Figure 2, but its degree distribution follows a power-law nature. As illustrated in Figure 3A, similar to Figure 2, in the high region of the CAN maximal conductance (${\bar{g}}_{C\,A\,N}>{\bar{g}}_{C\,A\,N,c}\approx 1.38$), increasing ${\bar{g}}_{C\,A\,N}$ progressively expands the moderate burst synchronization region to the left, covering a larger portion of the ${\bar{g}}_{N\,M\,D\,A}$ range; while in the low region of the CAN maximal conductance (${\bar{g}}_{C\,A\,N}<{\bar{g}}_{C\,A\,N,c}$), the burst asynchronization region occupies the entire ${\bar{g}}_{N\,M\,D\,A}$ range. Figure 3B shows the *R* distribution diagram for the Erdös-Rényi (ER) random network, which is constructed by connecting the existing DA neurons with an equal probability. Again, it is seen that in the high region of the CAN maximal conductance (${\bar{g}}_{C\,A\,N}>{\bar{g}}_{C\,A\,N,c}\approx 1.42$), the moderate burst synchronization region gradually expands to the left as ${\bar{g}}_{C\,A\,N}$ increases, covering a larger portion of the ${\bar{g}}_{N\,M\,D\,A}$ range; while in the low region of the CAN maximal conductance (${\bar{g}}_{C\,A\,N}<{\bar{g}}_{C\,A\,N,c}$), the burst asynchronization region occupies the entire ${\bar{g}}_{N\,M\,D\,A}$ range. Figures 2, 3 suggest that the main features of the *R* distribution diagram exhibit qualitatively similar trends across the tested topologies, suggesting the potential existence of a universal mechanism to explain all observed phenomena.

### 3.3 Inhibitory couplings participate in inducing synchronous bursts

**FIGURE 4:**
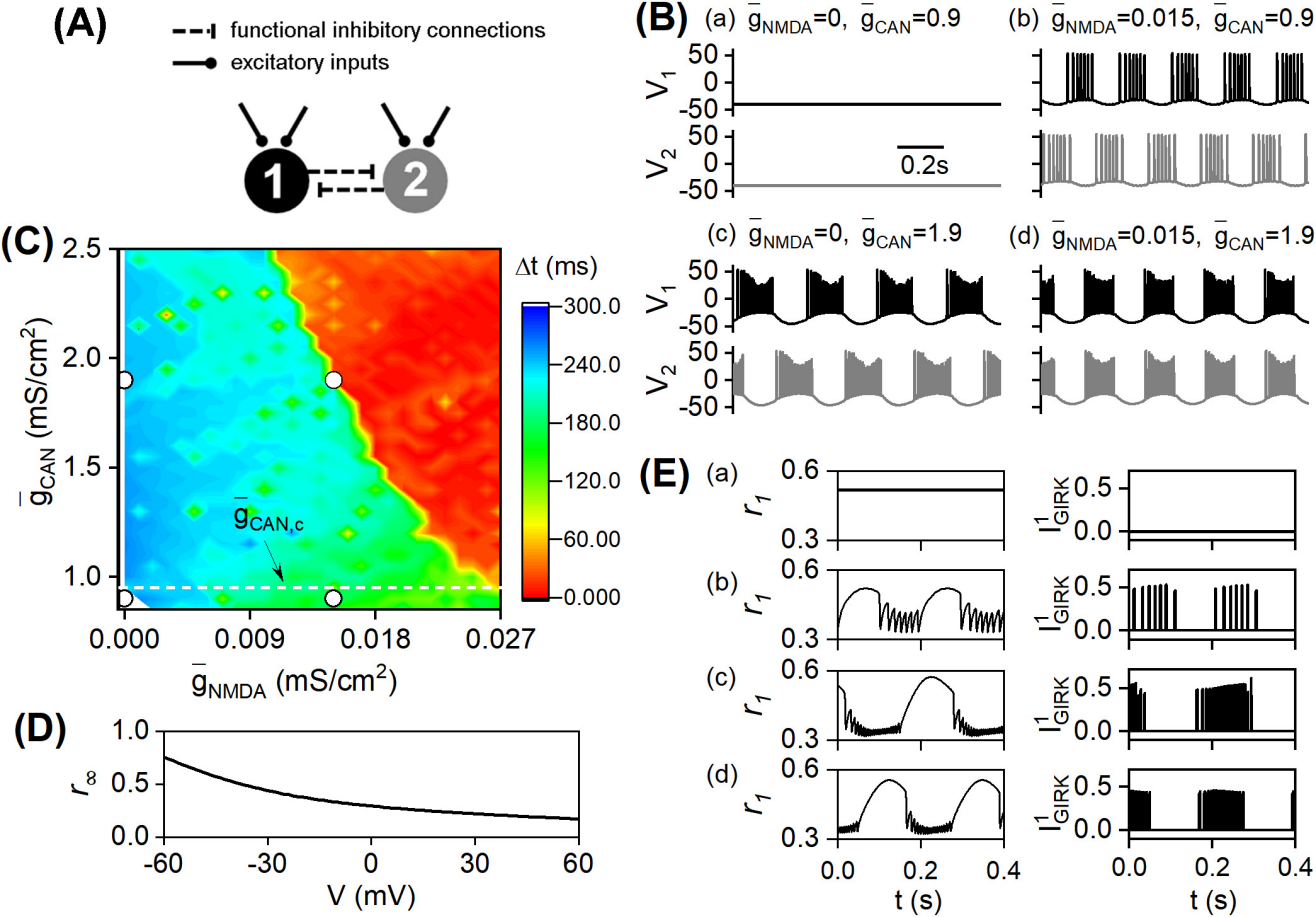
Synchronous burst induction in a dual-node DA network. **(A)** a dual-node DA network structure. **(B)** spatiotemporal dynamics. **(C)** synchronization index Δ*t* distribution. Inserted dots represent the Δ*t* values corresponding to the network dynamics shown in Figure 4B. The white dashed line denotes the critical CAN maximal conductance ${\bar{g}}_{C\,A\,N,c}$. **(D)** the steady activation curve *r*_∞_ for the gating variable *r*_1_ with respect to V. **(E)** the time evolution of *r*_1_ (Left) and the coupling current $I_{1}^{G\,I\,R\,K}$ (Right) corresponding to the spatiotemporal dynamics illustrated in Figure 4B.

During the induction of synchronous bursts, the network’s structure and connections stay constant; only the external excitatory inputs affecting the DA cells change. How does muscarinic receptor-modulated CAN current facilitate NMDA receptor-induced bursts synchronizing? To address this question, a simplified network is employed, which consists of two DA neurons and is also functionally interconnected via D2-GIRK currents, as illustrated in Figure 4A. The impacts of NMDA and CAN currents on spatiotemporal dynamics and synchronization index are re-evaluated in this dual-node network. The spatiotemporal dynamics are plotted in Figure 4B. Initially, as depicted in Figure 4Ba, the network remains at a resting state before muscarinic and NMDA receptor activation. Separately activating either receptor type drives the network into a burst asynchronization state (Figures 4Bb, Bc). However, simultaneous activation of both receptors leads the network into a burst synchronization state (Figure 4Bd). These results replicate the phenomena that muscarinic receptor-modulated CAN current promotes NMDA receptor-induced bursts synchronizing.

The average minimum time difference between bursts of coupled DA neurons, denoted as Δ*t*, is utilized to assess the synchronization of bursts in this dual-node network. A smaller Δ*t* value signifies greater network synchronization. As depicted in Figure 4C, the Δ*t* distribution diagram reproduces the main feature shown in Figures 2, 3. To be specific, in the high region of the CAN maximal conductance (${\bar{g}}_{C\,A\,N}>{\bar{g}}_{C\,A\,N,c}\approx 0.96$), as ${\bar{g}}_{C\,A\,N}$ increases, the burst synchronization region (Δ*t* < 60*ms*) progressively expands to the left, occupying a larger portion of the ${\bar{g}}_{N\,M\,D\,A}$ range; while in the low region of the CAN maximal conductance (${\bar{g}}_{C\,A\,N}<{\bar{g}}_{C\,A\,N,c}$), the burst asynchronization region (Δ*t* > 60*ms*) occupies the entire ${\bar{g}}_{N\,M\,D\,A}$ range. These findings reaffirm that higher levels of muscarinic receptor-modulated CAN current significantly increase the possibility of NMDA receptor-induced bursts synchronizing. The striking similarities between Figures 2–4 render that the dual-node network is ideal for exploring the mechanism by which diverse excitatory inputs synergistically induce synchronous bursts.

The effect of connections between DA neurons on these synchronous bursts is then analyzed. In order to monitor the changes in the couplings between DA neurons under different network dynamics, due to the symmetry of the dual-node network structure, the D2-GIRK current affecting neuron 1 ($I_{1}^{G\,I\,R\,K}$) is selected for observation. Assuming D2 receptors produce sufficient G protein, the gating variable *r*_1_ of $I_{1}^{G\,I\,R\,K}$ depends solely on neuron 1’s membrane potential *V*_1_. As depicted in Figure 4D, the steady activation curve for *r*_1_ decreases gradually with increasing *V*_1_. The analyses of $I_{1}^{G\,I\,R\,K}$ for different network dynamics are as follows. Prior to the activation of NMDA and muscarinic receptors, both DA neurons are in their resting state, with *V*_1_ and *V*_2_ at approximately −40*mV*, below the coupling threshold (Θ_*S*_ = −20*mV*). Therefore, *H*(*V*_2_) is 0, resulting in $I_{1}^{G\,I\,R\,K}$ being 0*pA*, despite a relatively high *r*_1_ (Figure 4Ea). After activation of NMDA or muscarinic receptors, burst firings occur, rendering *V*_2_ to exceed the coupling threshold, thus *H*(*V*_2_) equals 1. During this period, neuron 1 is in its *inter-burst* phase, with *V*_1_ typically below −40*mV*, resulting in a relatively large *r*_1_ and thus a high amplitude of $I_{1}^{G\,I\,R\,K}$ (Figures 4Eb, 4Ec). Simultaneous activation of NMDA and muscarinic receptors also triggers burst firings in neuron 2, making *H*(*V*_2_) equal 1. However, neuron 1 is in its *intra-burst* period, with *V*_1_ often above 0*mV*, leading to a comparatively low *r*_1_ and thus a marginally reduced amplitude of $I_{1}^{G\,I\,R\,K}$ (Figure 4Ed).

Theoretical studies on spiking networks have shown that sufficient inhibitory couplings, i.e., increases in inhibitory couplings, can improve the synchronization of resonators (Izhikevich, 2010; Chik et al., 2004). Our findings reveal that inhibitory coupling regulates the dynamics of bursting DA networks; However, compared to asynchronous states, its amplitude diminishes during synchronization, indicating that it is not the primary driver of synchronization. This prompts critical questions: If synchronization does not arise from inhibitory couplings, could it instead stem from the local dynamics of nodes? More specifically, might excitatory inputs drive a transition in burst modes—from integrators to resonators?

### 3.4 Transitioning burst modes from integrators to resonators leads to synchronization

The analysis then explores the properties of decoupled DA neurons to determine if they function as integrators or resonators. This is achieved by studying the bifurcation structure of equilibria during burst initiation, which involves two steps: identifying the discharge threshold *V*_*th*_ of the fast subsystem and conducting a phase-plane analysis within the reduced DA model.

To identify the discharge threshold *V*_*th*_ of the fast subsystem, the slow subsystem is removed away from the full DA model. *V*_*th*_ is then calculated within the remaining fast subsystem by adjusting the external DC current *I*_0_. The results reveal that *V*_*th*_ consistently stays around −38.7*mV*, regardless of variations in ${\bar{g}}_{N\,M\,D\,A}$.

**FIGURE 5:**
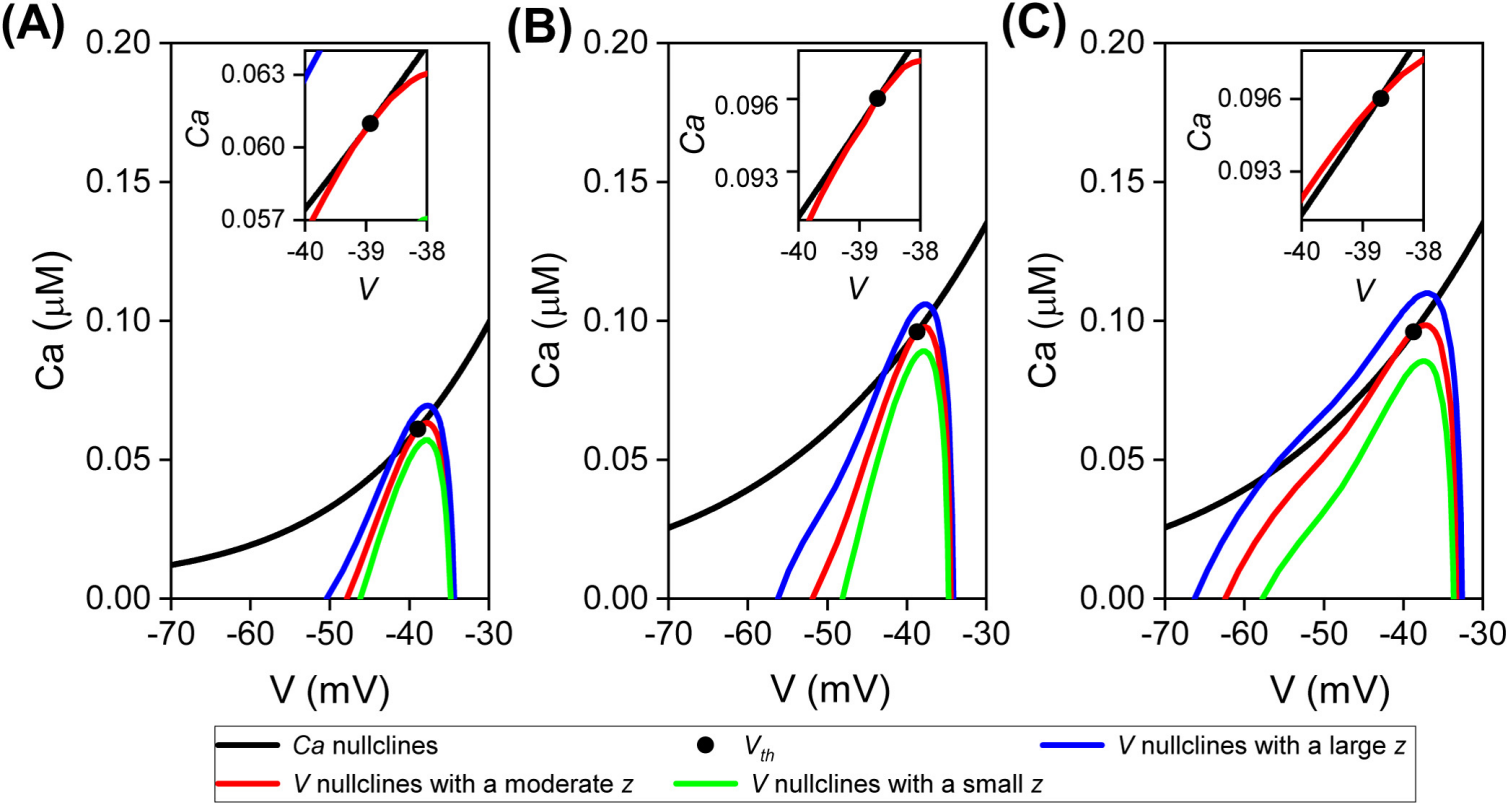
Phase-plane analysis within the reduced model of decoupled DA neuron. Analysis for each burst induced by NMDA receptor activation alone **(A)**, muscarinic receptor activation alone **(B)**, and simultaneous activation of both receptors **(C)**, respectively. *V* nullclines for large (blue), moderate (red), and small (green) *z*, along with *Ca* nullclines (black). Inserted graphs provide expanded views near the firing threshold of the fast subsystem *V*_*th*_ (black dots).

In the reduced DA model, conduct phase-plane analysis for each burst by setting *z* as the control parameter and calculating *Ca* nullclines and *V* nullclines. As shown in Figure 5, when *z* is large, the *V* nullclines (Figures 5A–C, blue curves) intersect the *Ca* nullclines (Figures 5A–C, black curves) at different voltages on both sides of *V*_*th*_ (Figures 5A–C, black dots). These intersections represent equilibria, with the right intersection being unstable and the left one stable. Therefore, the system resides at the left equilibrium, representing the resting state. A significant decrease in *z* results in a notable downward shift of the *V* nullclines (Figures 5A–C, green curves), preventing them from intersecting with the *Ca* nullclines in the region of interest. This change indicates the system has entered the firing state.

A moderate decrease in *z* leads to the intersection of *Ca* nullclines and *V* nullclines shifting to *V*_*th*_, marking the critical transition from a resting state to a firing state. This intersection at *V*_*th*_ varies across different scenarios. When NMDA and muscarinic receptors are activated separately, the *Ca* nullclines are tangent to the *V* nullclines (Figures 5A, B, red curves). However, simultaneous activation of both receptors results in the *Ca* nullclines intersecting the *V* nullclines (Figure 5C, red curve).

At *V*_*th*_, the tangency between the *Ca* nullclines and *V* nullclines signifies a SN bifurcation structure, confirming that bursts function as integrators. However, at *V*_*th*_, their intersection denotes an AH bifurcation, demonstrating that bursts act as resonators. This reveals that separately activating two receptors generates challenging-to-synchronize integrator bursts, while their simultaneous activation yields easy-to-synchronize resonator bursts. In summary, these findings illustrate that diverse excitatory inputs collaborate to induce synchronous bursts by transitioning burst behavior from integrator to resonator mode.

### 3.5 Sufficient Ca^2+^ accumulation leads to the transition of bursts

**FIGURE 6:**
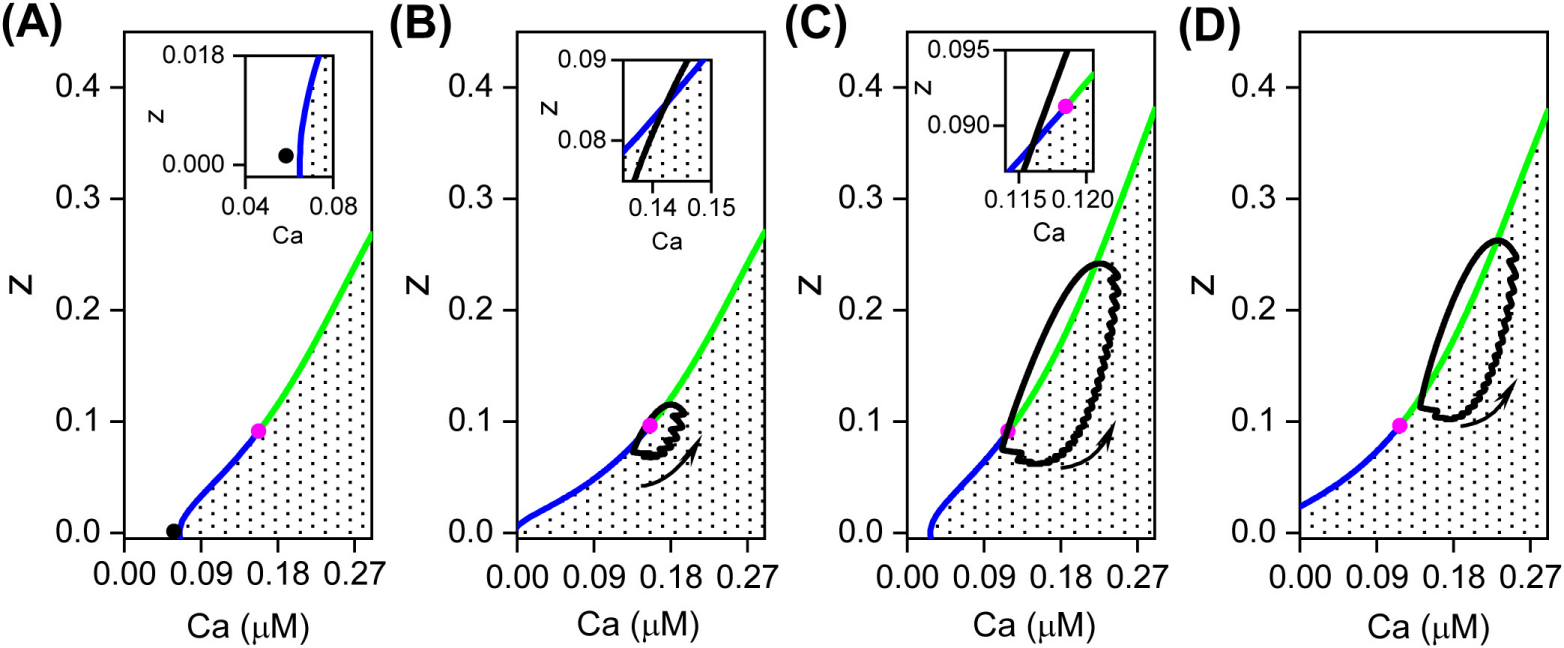
Slow-fast analysis within the full model of decoupled DA neuron. Analysis for each electrical activity under the following conditions: pre-activation of NMDA and muscarinic receptors **(A)**, NMDA receptor activation alone **(B)**, muscarinic receptor activation alone **(C)**, and simultaneous activation of both receptors **(D)**, respectively. The state-space distribution of *Ca* and *z* dynamics for each activity, alongside Saddle-Node (SN) curve (blue), Andronov-Hopf (AH) curve (green), and Bogdanov-Takens (BT) bifurcation (magenta dots).

To investigate how diverse excitatory inputs synergistically transition burst behavior to resonator mode, a fast-slow analysis within the full DA model is performed. For each electrical activity, the bifurcation curve of equilibria and the state-space distribution of *Ca* and *z* dynamics are plotted on the *Ca*-*z* plane. As shown in Figure 6, as *Ca* and *z* increase, the bifurcation curve of equilibria evolves from the SN curve (Figures 6A–D, blue curves) to the AH curve (Figures 6A–D, green curves) via a BT bifurcation (Figures 6A–D, magenta dots). These curves and bifurcations partition each *Ca*-*z* plane into a firing region (Figures 6A–D, shaded area) and a quiescent region (Figures 6A–D, unshaded area).

During examining the state-space distributions of *Ca* and *z* dynamics across different electrical activities, two key differences are identified. Firstly, there is a notable difference in form. Before activating NMDA and muscarinic receptors, the state-space distribution stabilizes at a point within the quiescent region of the *Ca*-*z* plane (Figure 6A, black dot). After activation, the distributions transition into trajectories oscillating between the quiescent and firing regions on the *Ca*-*z* plane (Figures 6B–D, black circles). A closer examination on each trajectory reveals that the burst terminates when Ca^2+^ accumulates enough to activate *z* of *I*_*SK*_, leading to the trajectory to intersect the AH curve at a higher *Ca*_*AH*_, thus entering the quiescent region and halting Ca^2+^ influx. The burst initiates when *Ca* drops sufficiently due to Ca^2+^ clearance, deactivating *z*, and resulting in the trajectory crossing the bifurcation curve at a lower *Ca*, thereby exiting the quiescent region and starting Ca^2+^ accumulation. Secondly, there is a vital difference in the bifurcation curves intersected by trajectories during burst initiation. When bursts are induced by the individual activation of two receptors, their trajectories intersect the SN curves (Figures 6B, C). However, when bursts are induced by the simultaneous activation of two receptors, the trajectory intersects the AH curve (Figure 6D).

To comprehend why trajectories intersect different bifurcation curves during burst initiation, it is essential to elucidate the relationship between *Ca* and *z*. As indicated in Equation (8), *z* is dependent on *Ca*, simplifying the slow subsystem to *Ca* alone. Previous studies (Chen et al., 2022) have shown that burst induction relies on a positive feedback loop between *Ca* and *I*_*CAN*_. Activating NMDA receptors supports this loop by directly providing Ca^2+^. Activating muscarinic receptors boosts the loop by increasing *I*_*CAN*_, causing depolarization and activating L-type Ca^2+^ channels, thereby indirectly supplying Ca^2+^. These mechanisms moderately boost *Ca* dynamics, respectively (Figures 6B, C). When both receptors are activated simultaneously, the combined direct and indirect Ca^2+^ significantly enhance *Ca* dynamics (Figure 6D). A moderate rise in *Ca* results in a corresponding moderate increase in *z*, while a significant rise in *Ca* leads to a substantial increase in *z*. Therefore, during burst initiation, a moderate increase in *Ca* and *z* dynamics typically causes the trajectory to intersect with the SN curve at a lower *Ca*_*SN*_, while a significant increase in *Ca* and *z* dynamics leads to the trajectory intersecting with the AH curve at a higher *Ca*_*AH*_.

In conclusion, sufficient Ca^2+^ accumulation leads to transitioning burst behavior from integrator to resonator mode. Simultaneous activation of both receptors is more effective in achieving the required Ca^2+^ accumulation compared to activating either receptor alone.

### 3.6 Stability of synchronization

When all the oscillators within DA neuronal networks are set at the synchronization manifold, they will remain synchronized. Now the crucial question is whether the synchronization manifold is stable in the presence of small perturbations.

**FIGURE 7:**
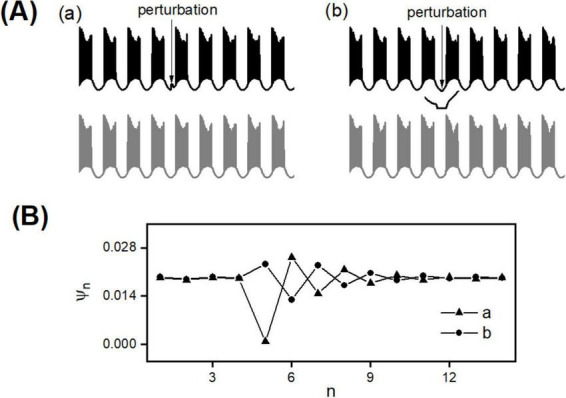
Synchronization stability in a dual-node DA network. Spatiotemporal dynamics under excitatory **(a)** and inhibitory **(b)** current pulse perturbations on neuron 1 **(A)**. Time evolution of phase difference Ψ_*n*_ **(B)**.

To address this question, in the two-node DA network, once the system has stabilized and achieved synchronization, neuron 1 is subjected to a current pulse *I*_0_ with a duration of 25 ms. When *I*_0_ = 2 pA, this represents an excitatory small perturbation (Figure 7Aa); when *I*_0_ = −2 pA, it represents an inhibitory small perturbation (Figure 7Ab). The occurrence times of the bursts, $t_{1}^{\left(n\right)}$ and $t_{2}^{\left(n\right)}$, are carefully recorded, and the phase difference between bursts of the coupled DA cells, Ψ_*n*_, is computed. As shown in Figure 7B, both excitatory and inhibitory perturbations induce changes in Ψ_*n*_; however, after several bursting periods, these changes decay oscillatively back to the pre-perturbation level (Figure 7B). These results strongly confirm the stability of burst synchronization induced by excitatory inputs.

**FIGURE 8:**
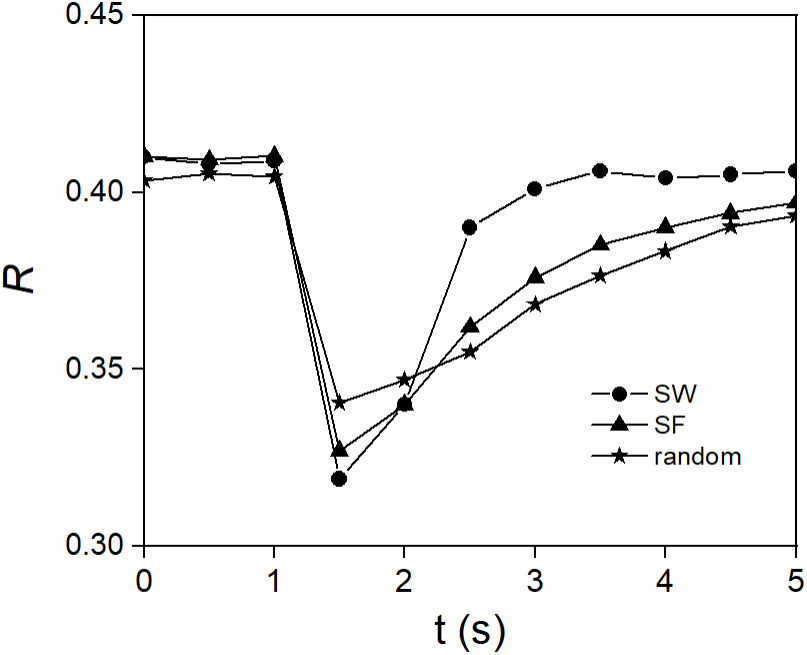
Synchronization stability in complex DA networks. The time evolution of *R* after random current pulse perturbations on 10 of the cells.

In complex networks composed of 50 DA neurons, after the systems reach moderate synchronized states, same current pulses, representing perturbations, are randomly introduced into 10 of the cells. The decay and recovery processes of the order parameter *R*(*t*) are then carefully monitored and recorded. As shown in Figure 8, overall, in all complex networks, *R*(*t*) exhibits a process of initial transient decay followed by gradual recovery. The subtle difference lies in that the relaxation time of *R*(*t*) in the SW network is significantly shorter than that in scale-free networks and random networks. These results further confirm that synchronization induced by excitatory inputs is stable, and such synchronization stability is more pronounced in SW networks.

## 4 Discussion

The midbrain DA input and output systems exhibit significant contrast. The input system receives diverse signals from multiple brain regions, with value-related information broadly distributed rather than confined to specific areas. However, the output system relies on the uniformity of bursts among DA cells to convey a clear signal to target regions. The transformation from diverse inputs to a uniform output is fundamental to DA’s function in the brain, with DA cell synchrony being the core mechanism driving this process. This study explores how diverse excitatory inputs lead to synchronized bursts. It reveals that activating NMDA and muscarinic receptors separately induces moderate intracellular Ca^2+^ accumulation, thereby resulting in integrator bursts and burst asynchronization. However, simultaneous activation of both receptors induces substantial Ca^2+^ accumulation, transitioning bursts to resonator behavior and thus prompting burst synchronization, which is universal. These findings indicate that diverse excitatory inputs from various brain regions collectively influence the characteristics of neighboring DA neurons, specifically their local dynamics, thereby inducing the synchronicity of the bursting DA network, and converting heterogeneous inputs into a homogeneous output.

Transitioning to resonator bursts is triggered by sufficient intracellular Ca^2+^ accumulation. Experimental evidences show that synchronous somato-dendritic (Lebowitz et al., 2023) and axonal (Banerjee et al., 2020) DA releases strongly depend on extensive and high-intensity Ca^2+^ oscillations. Ca^2+^ originates directly from NMDA receptors, voltage-gated Ca^2+^ channels (Liu et al., 2014), and Ca^2+^ stores (Zhu et al., 2004), as well as indirectly from G protein-linked receptors, such as mAChRs and mGluR1 (Prisco et al., 2002) by enhancing *I*_*CAN*_. It can be seen that modulating the intrinsic excitability and activating excitatory inputs both enhance the transition, thereby fostering synchronization.

In addition to resonator bursts, inhibitory connections mediated by D2-GIRK currents play a role in modulating synchronous bursts. In positive reinforcement paradigms, natural reward stimuli activate excitatory inputs, inducing bursts and upregulating D2 receptors (Kita et al., 2007; Jones and Fordahl, 2021). And, in negative reinforcement paradigms, aversive stimuli or cues activate GABAergic inhibitory inputs, inducing bursts through disinhibition (Lobb et al., 2011) and enhancing GIRK currents by activating GABA_*B*_ receptors (Condon et al., 2022). Theoretical studies on spiking networks suggest that an increase in inhibitory couplings can foster the synchronization of resonators (Izhikevich, 2010; Chik et al., 2004). This suggests that enhancing D2-GIRK currents through the upregulation of D2 receptors and the facilitation of GIRK currents, positive and negative reinforcement may promote the synchronization of excitatory input-induced and disinhibition-induced resonator bursts, respectively. Although the D2-GIRK current during synchronous bursts is slightly lower than that during asynchronous bursts, our subsequent studies have confirmed that a sufficient D2-GIRK current is also crucial for the induction of synchronous bursts. However, the synchronous stability promoted by the D2-GIRK current still requires further investigation.

The synchronization of bursts dictates phasic DA release, with the number of synchronous bursts affecting the amplitude of this release (Huang et al., 2025). During synchronous burst induction, excitatory inputs are complemented by disinhibition of inhibitory inputs, and disinhibition often leads to rebound depolarization. A moderate increase in rebound depolarization can directly trigger integrator bursts in some DA neurons, thereby facilitating the transition of bursts from integrator to resonator behavior in response to excitatory stimuli. This can modestly increase the number of synchronous bursts and reasonably enhance the amplitude of phasic DA release, thereby boosting reward responses. A significant rise in rebound depolarization may directly induce resonator bursts, causing numerous DA neurons to emit resonator bursts in response to excitatory stimuli. This can result in an excessive increase in the number of synchronous bursts and an abnormal rise in the amplitude of phasic release, potentially leading to addictive responses. Enhancing inhibitory inputs and upregulating channels responsible for rebound current can both amplify rebound depolarization. Adolescent DA neurons exhibit higher GABA tone compared to adults (Iacino et al., 2024), resulting in heightened reward responses in adolescents (Braams et al., 2015). Nicotine exposure can impair GABA_*A*_ receptor signaling in VTA-GABA neurons, causing excessive excitation of these neurons and increased GABAergic projections to VTA-DA neurons, thereby promoting the consumption of substances such as alcohol (Thomas et al., 2018) and diazepam (Ostroumov et al., 2020). Moreover, addictive drugs like cocaine and morphine not only enhance GABAergic projections to VTA-DA neurons (Weitz et al., 2021; Wang et al., 2023) but also up-regulate HCN channels in these neurons (Mu et al., 2023; Yin et al., 2024), collectively exacerbating addiction. Although multiple experimental results provide supportive evidence for the aforementioned speculations, the mechanism by which rebound depolarization promotes the formation of synchronous bursts and the stability of such synchronization still require further in-depth exploration.

## Data Availability

The original contributions presented in this study are included in this article/supplementary material, further inquiries can be directed to the corresponding author.
